# Gender and age disparity in the initiation of life-supporting treatments: a population-based cohort study

**DOI:** 10.1186/s12910-017-0222-9

**Published:** 2017-11-15

**Authors:** Peng-Sheng Ting, Likwang Chen, Wei-Chih Yang, Tien-Shang Huang, Chau-Chung Wu, Yen-Yuan Chen

**Affiliations:** 1 Department of Medicine, National Taiwan University College of Medicine, Taipei, Taiwan; 2 Institute of Population Health Sciences, National Health Research Institutes, Miaoli, Taiwan; 3 Department of Medical Education, Cathay General Hospital, Miaoli, Taiwan; 4 Graduate Institute of Medical Education and Bioethics, National Taiwan University College of Medicine, Taipei, Taiwan; 5 Department of Internal Medicine, National Taiwan University Hospital, Taipei, Taiwan; 6 Department of Medical Education, National Taiwan University Hospital, Taipei, Taiwan

**Keywords:** Extracorporeal membrane oxygenation, Cardiopulmonary resuscitation, Life-supporting treatment, Trend, Disparity

## Abstract

**Background:**

The relationships between age and the life-supporting treatments use, and between gender and the life-supporting treatments use are still controversial. Using extracorporeal membrane oxygenation as an example of life-supporting treatments, the objectives of this study were: (1) to examine the relationship between age and the extracorporeal membrane oxygenation use; (2) to examine the relationship between age and the extracorporeal membrane oxygenation use; and (3) to deliberate the ethical and societal implications of age and gender disparities in the initiation of extracorporeal membrane oxygenation.

**Methods:**

This is a population-based, retrospective cohort study. Taiwan’s extracorporeal membrane oxygenation cases from 2000 to 2010 were collected. The annual incidence rate of extracorporeal membrane oxygenation use adjusting for both age and gender distribution for each year from 2000 to 2010 was derived using the population of 2000 as the reference population. The trend of extracorporeal membrane oxygenation use was examined using time-series linear regression analysis. We conducted joinpoint regression for estimating the trend change of extracorporeal membrane oxygenation use.

**Results:**

The trends of extracorporeal membrane oxygenation use both for different gender groups, and for different age groups have been significantly increasing over time. Men were more likely to be supported by extracorporeal membrane oxygenation than women. Women’s perspectives toward life and death, and women’s perception of well-being may be associated with the phenomenon. In addition, the patients at the age of 65 or older were more likely to be supported by extracorporeal membrane oxygenation than those younger than 65. Family autonomy/family-determination, and the Confucian tradition of filial piety and respecting elders may account for this phenomenon.

**Conclusions:**

This study showed gender and age disparities in the initiation of extracorporeal membrane oxygenation use in Taiwan, which may be accounted for by the cultural and societal values in Taiwan. For a healthcare professional who deals with patients’/family members’ medical decision-making to initiate life-supporting treatments, he/she should be sensitive not only to the legality, but also the societal and ethical issues involved.

## Background

Extracorporeal membrane oxygenation (ECMO) is a technique that uses a modified heart-lung machine to support the severely ill patients for several days or weeks as a bridge to further treatment or while awaiting recovery during cardiac or respiratory failure. Until now, the indications of initiating ECMO are defined as respiratory failure or cardiac failure refractory to conventional intensive care [1–4].

According to Extracorporeal Life Support Registry Report, a collection of ECMO cases reported to Extracorporeal Life Support Organization by hundreds of medical institutions worldwide which operated ECMO, the annual number of ECMO patients gradually rose since 1990. ECMO is mostly used to support neonates who, compared to adults and pediatric patients, not only have the highest rates of surviving ECMO use, but also surviving to hospital discharge [5].

Given that ECMO use to support patients is rapidly increasing [5, 6], examining the issues in relation to ECMO use is attracting more and more attention. Most studies on ECMO have focused on outcomes [7–12], and several of them reported that approximately 48% to 52% of patients supported by ECMO were male [7–9, 13]. In this study, it is paramount to examine gender and age disparities in ECMO use. The objectives of this study were as follows: (1) to examine the gender disparities in ECMO use; (2) to examine the age disparities in ECMO use; (3) to estimate the trend of ECMO use as stratified by gender; (4) to estimate the trend of ECMO use as stratified by age; (5) to examine the trend change of ECMO use for each gender and age group; and (6) to deliberate the ethical, cultural, and societal implications of the study results.

## Methods

### Data collection

We obtained secondary data from the Taiwan National Health Insurance Research Dataset (NHIRD) by using SAS 9.3 software (for Windows PC) to build our analytical data file. We extracted and organized registration and hospital-care data for all patients supported by ECMO for any length of time between January 1, 2000 and December 31, 2010. We used person-year to estimate the incidence rate of ECMO use. We estimated the annual number of person-years in Taiwan for each year from 2000 to 2010.

### Direct standardization

Because the gender and age distribution in the population changes each year, the population of Taiwan in the year 2000 is used as the reference population in conducting both direct age standardization and direct gender standardization.

The annual incidence rate (IR) of ECMO use adjusting for both age and gender distribution for each year from 2000 to 2010 was derived using the population of 2000 as the reference population. We defined the gender-specific age-adjusted incidence rate of ECMO use (GIR) as the annual number of ECMO cases per million person-years adjusting for age distribution using direct age standardization. The difference in ECMO use between gender groups was then examined by comparing the trend of the GIR for men with that for women. In addition, we defined the age-specific gender-adjusted incidence rate of ECMO use (AIR) as the annual number of ECMO cases per million person-years adjusting for gender distribution using direct gender standardization. We compared the trends of the AIR for each age group: Neonates, aged 30 days and younger; Pediatrics, from the age of 31 days to 17 years old; Adults, from the age of 18 years old to 64 years old; Elders, aged 65 years and older.

### Statistical analyses

We fitted time-series linear regression models to investigate whether the trend of ECMO use was significantly increasing over time. For estimating the trend change, we performed joinpoint regression using Joinpoint Regression Program 3.5.2 for Windows PC [14]. We supplied the minimum and the maximum number of joinpoints as zero and four respectively, the minimal number of observations from a joinpoint to either end of the data as two, and the minimal number of observations between two joinpoints as one. Results with a *p* value of less than or equal to .05 were regarded as statistically significant. All statistical analyses in this study were carried out using the software package of STATA MP 11.0 for Windows PC.

## Results

The total number of ECMO cases in Taiwan from 2000 to 2010 was 6099. The annual total number of ECMO cases approximately quadrupled, from 257 cases in 2000 to 1104 cases in 2010. The annual IR of ECMO use in Taiwan approximately quadrupled, from 11.87 cases per million person-year in 2000 to 44.36 cases per million person-year in 2010. The time-series linear regression analysis was used to estimate the trend of the annual IR, and showed that ECMO use in Taiwan has significantly increased over time (*p* < .01) with the adjusted R-square of 0.78 (Table 1).

**Table 1:** Extracorporeal membrane oxygenation uses in Taiwan from 2000 to 2010

Year	Case	Non-standardized incidence rate^a^	Standardized incidence rate^b^
2000	257	11.87	11.87
2001	274	12.40	12.35
2002	311	13.97	13.95
2003	461	20.58	20.43
2004	435	19.22	18.86
2005	393	17.13	16.89
2006	413	17.87	17.59
2007	666	28.62	28.03
2008	857	36.55	35.43
2009	886	37.68	38.35
2010	1104	46.74	44.36

^a^“Non-standardized incidence rate” means the annual number of ECMO cases per million person-year in that year

^b^“Standardized incidence rate” means the annual number of ECMO cases per million person-year adjusting for the age and gender distribution in 2000

### Extracorporeal membrane oxygenation use between genders

**Fig. 1:**
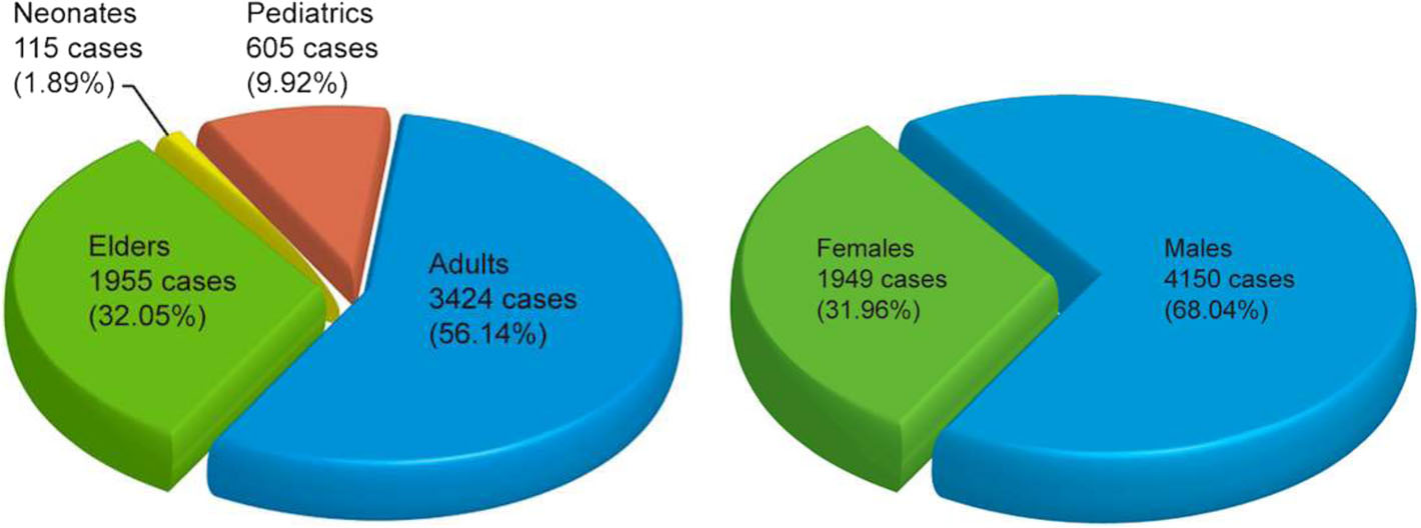
The total number and proportion of each age and gender group of extracorporeal membrane oxygenation uses in Taiwan

A total of 1949 (31.96%) of all ECMO cases were females (Fig. 1). The number of female ECMO cases gradually increased from 87 cases in 2000 to 343 cases in 2010. By comparison, the number of male ECMO cases gradually increased from 170 cases in 2000 to 761 cases in 2010. As indicated by the GIR, the male GIR increased from 15.50 cases per million person-year in 2000 to 61.38 cases per million person-year in 2010, while the female GIR increased from 8.19 cases per million person-year in 2000 to 26.98 cases per million person-year in 2010. The male GIR to the female GIR ratio was 1.89 and 2.27 in 2000 and 2010 respectively, with the lowest ratio of 1.66 in 2001 and the highest ratio of 2.54 in 2003 (Table 2).

**Table 2:** The comparison of extracorporeal membrane oxygenation uses between different gender groups

	Case		Incidence	
	Male	Female	Male	Female
Year	N	N	GIR^a^	GIR^a^
2000	170	87	15.50	8.19
2001	173	100	15.37	9.28
2002	211	100	18.62	9.12
2003	331	128	29.12	11.47
2004	291	141	25.27	12.28
2005	250	142	21.34	12.50
2006	276	137	23.58	11.42
2007	451	215	37.97	17.88
2008	585	272	48.45	21.96
2009	651	284	53.21	23.19
2010	761	343	61.38	26.98

^a^GIR denotes for “gender-specific age-adjusted incidence rate of extracorporeal membrane oxygenation,” which means the annual number of ECMO cases per million person-year adjusting for age distribution in 2000

The gender status of some ECMO users in the NHIRD is missing: one in 2001, two in 2003, three in 2004, and one in 2004

**Fig. 2:**
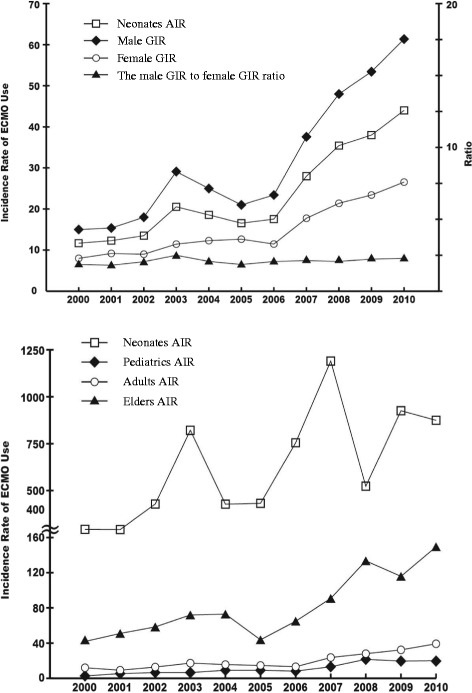
The trend of each age and gender group of extracorporeal membrane oxygenation uses from 2000 to 2010 in Taiwan

We identified that the trend of the male GIR was significantly increasing since 2000 (β coefficient = 4.42, *p* < .01, adjusted R-square = 0.81). The trend of the female GIR was also significantly increasing over time (β coefficient = 1.82, *p* < .01, adjusted R-square = 0.85). However, the male GIR to the female GIR ratio demonstrated a yearly increment of 0.04, which is not statistically significant (*p* = .13) (Fig. 2).

Joinpoint regression for the trend of the male GIR and the trend of the female GIR finally selected the model with one joinpoint in 2006 as the best-fit models. In comparison, the best-fit model for the male GIR to the female GIR ratio selected by joinpoint regression was the model with no joinpoint (Table 3).

**Table 3:** Joinpoint Regression Analysis for the trends of extracorporeal membrane oxygenation uses over time

	Joinpoint	Pre-joinpoint slope	Post-joinpoint slope
IR^a^	One joinpoint at 2006	1.34	6.27
Female GIR^b^	One joinpoint at 2006	0.84	3.54
Male GIR	One joinpoint at 2006	1.84	8.94
M/F ratio^c^	No joinpoint	Not applicable	Not applicable
Neonate AIR^d^	No joinpoint	Not applicable	Not applicable
Pediatric AIR	No joinpoint	Not applicable	Not applicable
Adult AIR	One joinpoint at 2006	1.13	5.90
Elder AIR	No joinpoint	Not applicable	Not applicable

^a^IR denotes for “the incidence rate of extracorporeal membrane oxygenation use adjusting for gender and age”

^b^GIR denotes for “gender-specific age-adjusted incidence rate of extracorporeal membrane oxygenation use”

^c^M/F ratio denotes for “the male GIR to female GIR ratio”

^d^AIR denotes for “age-specific gender-adjusted incidence rate of extracorporeal membrane oxygenation use”

### Extracorporeal membrane oxygenation use between age groups

A total of 115 (1.89%), 605 (9.92%), 3424 (56.14%), and 1955 (32.05%) of all ECMO cases were Neonates, Pediatrics, Adults, and Elders, respectively (Fig. 1). The number of Neonates, Pediatrics, Adults and Elders ECMO cases gradually increased from 8, 25, 142, and 82 cases in 2000 to 11, 79, 644, and 370 cases in 2010, respectively. As indicated by the AIR, Neonates AIR increased from 330.98 cases per million person-year in 2000 to 887.44 cases per million person-year in 2010. Pediatrics AIR increased from 4.47 cases per million person-year in 2000 to 17.13 cases per million person-year in 2010. Adults AIR increased from 10.12 cases per million person-year in 2000 to 39.58 cases per million person-year in 2010. Elders AIR increased from 44.39 cases per million person-year in 2000 to 157.39 cases per million person-year in 2010 (Table 4).

**Table 4:** The comparison of extracorporeal membrane oxygenation uses between different age groups

	Case				Incidence			
	Younger than 30 d/o^a^	31 d/o— 17 y/o^b^	18 y/o— 64 y/o	Older than 65 years	Younger than 30 d/o	31 d/o— 17 y/o	18 y/o— 64 y/o	Older than 65 years
Year	N	N	N	N	AIR^c^	AIR	AIR	AIR
2000	8	25	142	82	330.98	4.47	10.12	44.39
2001	6	35	138	94	294.36	6.33	9.51	50.34
2002	8	35	155	113	428.51	6.41	10.55	58.77
2003	15	42	261	141	844.33	7.80	17.59	71.31
2004	7	49	230	146	425.53	9.33	15.25	72.11
2005	7	50	239	96	430.93	9.74	15.55	46.01
2006	12	44	215	142	752.59	8.53	13.81	66.09
2007	19	63	383	201	1197.19	12.58	24.30	91.18
2008	8	102	451	296	523.99	20.77	28.20	131.55
2009	14	81	566	274	930.88	17.11	35.17	119.39
2010	11	79	644	370	887.44	17.13	39.58	157.39

^a^d/o denotes for “days old”

^b^y/o denotes for “years old”

^c^AIR denotes for “age-specific gender-adjusted incidence rate of extracorporeal membrane oxygenation,” which means the annual number of ECMO cases per million person-year adjusting for the gender distribution in 2000

We used time-series linear regression analysis to examine the trends of Neonates AIR, Pediatrics AIR, Adults AIR, and Elders AIR. We found that all of the four trends of AIR were significantly increasing over time, with *p* values of .02 (β coefficient = 60.44), <.01 (β coefficient = 1.44), <.01 (β coefficient = 2.86), <.01 (β coefficient = 9.94) for Neonates AIR, Pediatrics AIR, Adults AIR and Elders AIR, respectively. (Fig. 2).

Joinpoint regression for the trend of Adult AIR finally selected the model with one joinpoint in 2006 as the best-fit model. In comparison, the best-fit model for the trend of Neonates AIR, Pediatrics AIR and Elders AIR selected by Joinpoint Regression was the model with no joinpoint (Table 3).

## Discussion

### Main findings

An examination of gender and age disparities of ECMO use demonstrated that both the numbers of the male and female ECMO cases have dramatically increased since 2000. Male patients were more likely to be supported by ECMO than female patients, with the male GIR to the female GIR ratio ranging from 1.69 to 2.57. In addition, Neonates AIR, Pediatrics AIR, Adults AIR and Elders AIR have significantly increased since 2000. Among the four age groups, patients aged 65 years and older (Elders) were much more likely than patients aged from 31 days to 17 years (Pediatrics), and from 18 years to 64 years (Adults) to be supported by ECMO. Particularly, the trends of the male GIR, the female GIR and Adult AIR all had a joinpoint in 2006, implying a significant trend change of ECMO use following 2006.

### Gender disparity in extracorporeal membrane oxygenation use

It is common that male patients receive more life-supporting treatments (LSTs) than female patients. Goodlin et al. reported that, based on SUPPORT dataset (the Study to Understand Prognoses and Preferences for Outcomes and Risks of Treatments), male patients were more likely to receive cardiopulmonary resuscitation (CPR) than female patients with an odds ratio of 1.39 [15]. Ahn et al. examined the relationship between gender and CPR efforts, and found that only 37% of resuscitated patients were female [16]. Some studies tried to explain these results by examining women’s attitudes toward LSTs use.

A study, using hypothetical scenarios, showed that women expressed a significantly weaker will to live than men, as well as less desire to prolong life by LSTs in all the scenarios presented to them [17]. Bookwala et al. examined gender differences in the preferences for LSTs in response to various health state scenarios, and suggested that men preferred LSTs more than women, while women showed a greater desire for death with dignity and better quality of life than men [18]. These studies, based on both actual cases and hypothetical scenarios, demonstrated that women receive fewer LSTs, or prefer to receive fewer LSTs than men.

Our study results are consistent with the cross-cultural phenomenon that women receive fewer LSTs than men. However, the differences between gender groups in receiving LSTs as indicated by ECMO use is much more profound in Taiwan than those reported by the rest of the world. Several reasons may account for the remarkable difference in gender disparities of ECMO use.

Women’s perspectives toward life and death may account for why women receive fewer LSTs. As proposed by Carmel, women usually play a more paramount role than men as a care-giver when their family members are ill, and therefore have much more experience being exposed to aggressive LSTs to extend life and severely compromised quality of life for those being supported by LSTs. Women therefore are more likely to accept the limitations of medical interventions to support life. Encountering more critical illness, women may be less likely than men to insist on LSTs to extend life [17]. Therefore, the different life experiences between men and women may account for the gender disparity in ECMO use in Taiwan.

Women’s perception of well-being is also a crucial factor associated with their less willingness to request LSTs. Gender-based difference in well-being has been reported by many social scientists. They stated that, although women live obviously longer than men, they are socially and psychologically disadvantaged on most indicators of well-being [19, 20]. As a result, when encountering critical illness, they are less likely to request LSTs than men.

In addition, women’s self-perceived burden to others may explain the gender disparity in LSTs use as indicated by ECMO. According to a study conducted by Tang et al., women were more likely to perceive themselves as a burden to others in face of end-of-life decision-making than men were [21]. Thus, women would be more likely to consent to a DNR order for relieving their self-perceived burden to others.

### Age disparity in extracorporeal membrane oxygenation use

Several studies have demonstrated that age is associated with the use of LSTs. Goodlin et al. reported that older patients were less likely to be resuscitated [15]. Brandberg et al. found that patients above 80 years old received fewer invasive interventions compared with patients aged between 65 and 79 [22]. Another study conducted by Reignier et al. showed that patient age is an independent predictor of the medical decision to forgo LSTs [23]. All these studies conducted in Western societies consistently showed that older age was associated with fewer LSTs provided to patients.

Based on a study to examine the elderly persons’ wishes, Carmel et al. argued that older age was associated with fewer LSTs. They concluded that the most powerful factor to predict patient’s choice regarding the use of LSTs is the patient’s personal experience with other people’s illnesses. Accompanied with the fears of death and dying, the results of such experiences, whether positive or negative, influence the use of LSTs on the same direction. Because personal experiences with other people’s critical illnesses are mostly negative, a person with more personal experiences with other people’s illnesses is generally less likely to support his/her own life with LSTs [24]. Despite being a convincing argument, it is not evident for the current practice of ECMO in Taiwan.

According to Extracorporeal Life Support Registry Report International Summary January 2017, 44.78% of reported worldwide ECMO cases were neonates, and 31.07% of them were adults and older [5]. In comparison, only 115 (1.88%) ECMO cases were neonates, and 1955 (32.05%) ECMO cases were elders in Taiwan from 2000 to 2010 (Fig. 1), which are so different from those have been reported by Extracorporeal Life Support Registry Report International Summary January 2017. Our findings contradict many studies which clearly reported that older patients tend to receive fewer LSTs [25–27]. Several reasons may account for this unique situation:

First, individual autonomy/self-determination and family autonomy/family-determination: Western societies mostly honor individual autonomy/self-determination, while Taiwan, as part of East Asian societies where Confucianism is influential, mostly values family autonomy/family-determination. Fan and Lee both proposed the theoretical foundations, based on Confucianism, in support of family autonomy/family-determination in the EastAsian societies, and also highlighted the distinctions between individual autonomy/self-determination and family autonomy/family-determination [28, 29]. Although Chen et al. had reported that the Western societal value of individual autonomy/self-determination has been more and more influential, the East Asian societal value of family autonomy/family-determination undoubtedly still remain more influential [30]. In addition, surrogate decision-making by family members is more prevalent in the initiation of ECMO than the initiation of other LSTs [31], because patients, suffering from cardiac or respiratory failure refractory to conventional treatment, are usually incapable of autonomous decision-making. Therefore, a patient’s self-determination, usually influenced by his/her personal experiences with other people’s critical illness as proposed by Carmel et al., plays little role in determining whether to receive LSTs or not. Especially when patients are imminently dying, family members as the surrogate decision-makers tend to overestimate patient preference for the use of LSTs [32], and in Taiwan, family members usually have poor-to-fair consensus on LST-related issues [33].

Second, the Confucian tradition of filial piety and respecting elders: Taiwan, as well as other East Asian countries, is deeply influenced by Confucianism. One of the core values highlighted by Confucianism is filial piety and respect for elders. Therefore, when the elder is imminently dying, family members are more likely to request LSTs for the elder as a token of their filial piety and respect for the elder than the family members in Western societies in a similar situation. In addition, to avoid being labeled as having no filial piety and no respect for elders, family members are unlikely to request withholding or withdrawing LSTs, even when they may realize that withholding or withdrawing LSTs for the elder is ethically appropriate.

### Trend change in extracorporeal membrane oxygenation use

Prior studies have proposed the possibility that the significantly increased ECMO uses in Taiwan’s population might be associated with significant social events [6] and their related over-optimistic reports on the Internet and newspapers [34]. We also highlighted the possibility that the media literacy of the audience, i.e. patients, family members, and health care workers, might play an important role on medical decision-making, as well as decision-making to initiate, withhold, or withdraw LSTs [35]. By further stratifying into different sub-populations, we found that the ECMO use was significantly increased following 2006 in both gender groups and in the adult population. Therefore, the decision-making of both gender groups and the adult population to initiate ECMO might be influenced by information from the media. How the media literacy of an individual associates with LSTs decision-making, as well as other medical decision-making, should be further investigated in the future.

By studying gender and age disparities in LSTs as represented by ECMO use, we found that men were more likely than women to be supported by ECMO, which can be partly explained as the result of patriarchy, gender discrimination towards women in Taiwan, women’s negative life experiences with critical illness, as well as women’s perceptions of well-being in a society. In addition, elderly patients in Taiwan were more likely to be supported by ECMO than in the rest of the world, which could reflect the adherence to the core values of filial piety and respect for the elders. Our study results suggest that patients’/family members’ medical decision-making to request the use of LSTs is highly associated with the societal and cultural values of the particular context. Therefore, for a healthcare professional who deals with patients’/family members’ medical decision-making to initiate, withhold or withdraw LSTs, he/she should be sensitive not only to the legality, but also the societal and cultural values involved.

### Strengths and limitations

By obtaining nationwide data, we were able to firstly examine the gender and age disparities of ECMO use in this population-based study. This study accurately estimated the annual ECMO use per million person-years for gender and age groups using direct standardization, and the population of Taiwan in the year 2000 as the reference population in conducting both direct age standardization and direct gender standardization.

Our study also has a number of limitations. First, this study was conducted in Taiwan, which has the highest prevalence rate of ECMO use in the world. The external generalizability of the results might be of concerns. Second, ECMO use was identified relying exclusively on the Taiwan NHIRD, which is reliable, but not completely so. Nevertheless, our sample size is so large that the missing data and few unreliable data are negligible, and does not obscure the implication of the study results. Third, given that this is a population-based, retrospective study, we would never know whether the criteria for initiating ECMO use were strictly followed in all cases included in this study.

## Conclusion

Taiwan has become the country with the most prevalent ECMO use in the world. We examined the current medical practice of ECMO with a particular focus on the differences between gender and age groups, as well as the trend change of ECMO use in different groups. We identified the disparities in ECMO use in different gender and age groups, and also the increasing ECMO use following 2006 in both gender groups and the adult group. The study results suggested that patients’/family members’ medical decision-making to request LSTs is associated with the ethical, cultural, and societal values surrounding the particular context. Future studies may be focused on further examining the ethical, cultural, and societal implications related to the gender and age disparities of ECMO use in Taiwan using qualitative research methods.

## Abbreviations

AIR: The age-specific gender-adjusted incidence rate of ECMO use
ECMO: Extracorporeal membrane oxygenation
GIR: The gender-specific age-adjusted incidence rate of ECMO use
IR: The incidence rate of ECMO use
NHIRD: National Health Insurance Research Dataset
